# Equivalent input produces different output in the UniFrac significance test

**DOI:** 10.1186/1471-2105-15-278

**Published:** 2014-08-13

**Authors:** Jeffrey R Long, Vanessa Pittet, Brett Trost, Qingxiang Yan, David Vickers, Monique Haakensen, Anthony Kusalik

**Affiliations:** Department of Computer Science, University of Saskatchewan, 176 Thorvaldson Bldg, 110 Science Place, Saskatoon, Canada; Contango Strategies Ltd, LFK Biotechnology Complex, 15-410 Downey Road, Saskatoon, Canada; Department of Mathematics and Statistics, University of Saskatchewan, 106 Wiggins Road, Saskatoon, Canada; Department of Pathology and Laboratory Medicine, University of Saskatchewan, 106 Wiggins Road, Saskatoon, Canada

**Keywords:** Unifrac, Computer methodologies, Metagenomics

## Abstract

**Background:**

UniFrac is a well-known tool for comparing microbial communities and assessing statistically significant differences between communities. In this paper we identify a discrepancy in the UniFrac methodology that causes semantically equivalent inputs to produce different outputs in tests of statistical significance.

**Results:**

The phylogenetic trees that are input into UniFrac may or may not contain abundance counts. An isomorphic transform can be defined that will convert trees between these two formats without altering the semantic meaning of the trees. UniFrac produces different outputs for these equivalent forms of the same input tree. This is illustrated using metagenomics data from a lake sediment study.

**Conclusions:**

Results from the UniFrac tool can vary greatly for the same input depending on the arbitrary choice of input format. Practitioners should be aware of this issue and use the tool with caution to ensure consistency and validity in their analyses. We provide a script to transform inputs between equivalent formats to help researchers achieve this consistency.

**Electronic supplementary material:**

The online version of this article (doi:10.1186/1471-2105-15-278) contains supplementary material, which is available to authorized users.

## Background

UniFrac is a tool for comparing microbial communities while taking phylogenetic distance between organisms into account. It is available as a web service hosted by the University of Colorado at Boulder at http://bmf2.colorado.edu/unifrac/index.psp, and is described by Lozupone, Hamady and Knight [1, 2]. According to recent work by the authors, UniFrac is a popular tool that has been cited in over 150 publications [3].

**Figure 1:**
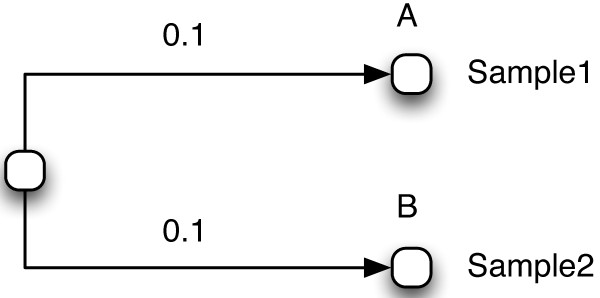
**Simple input tree for UniFrac.** Simple example of UniFrac input that does not use abundance counts.

UniFrac has two main uses that are relevant to this paper. The first use is to calculate the UniFrac distance between communities, which is done by considering the branches of an input phylogenetic tree that are unique to one community or the other. The second use is to determine whether two communities are different from each other with statistical significance, taking into account the phylogenetic information implied by the UniFrac distance metric. Briefly, UniFrac uses a phylogenetic tree as input, along with sample labels at each leaf node of the tree. To perform tests of significance, UniFrac creates new trees by randomly re-assigning the sample labels. For each random re-assignment, it calculates whether the resulting UniFrac distance between samples is equal to or greater than the distance between samples in the original labeling. The p-value reported is the number of random labelings that have an equal or greater distance between samples divided by the total number of randomized labelings.In Figure 1 we show a very simple example of UniFrac input. It consists of a tree having only two leaf nodes, with an arbitrary distance value of 0.1 on the branch leading to each leaf. In this example, the organism represented by node A was found in Sample1 and the organism represented by node B was found in Sample2. Uploading this data to the UniFrac web tool and performing the "UniFrac Significance Test" gives a p-value of 1.0. This means that in 100% of the randomly labelled trees, the UniFrac distance between Samples 1 and 2 was equal to or greater than the UniFrac distance between the two samples in the original tree, and thus the two environments are not different with statistical significance. We can see why this is the case through the following reasoning. A random assignment of the labels will assign the Sample1 label to either node A or B and Sample2 to the other. Since the tree is symmetric, the UniFrac distance between Sample 1 and 2 will be the same in either scenario.

**Figure 2:**
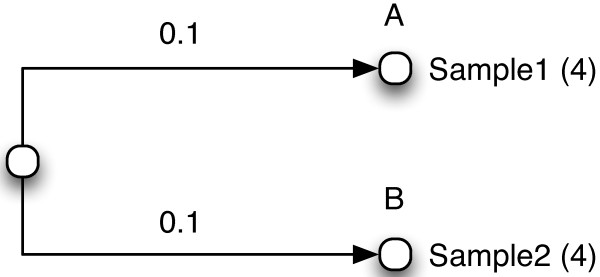
**Input tree for UniFrac with abundance counts.** A tree input into UniFrac where abundance counts are included at each leaf node in compact form.

UniFrac also allows for abundance counts to be included in the input data. For instance, in addition to indicating that node A was found in Sample1, we can also indicate the number of times it was found. Figure 2 shows a tree where node A was found 4 times in Sample1 and node B was found 4 times in Sample2. It is also possible for more than one sample label to be associated with each leaf node, and for each such label to have its own abundance count. The UniFrac web tool has an option to use these abundance counts when calculating the UniFrac distance between samples by using the *weighted UniFrac* algorithm [2].

It is exclusively the weighted version of the algorithm that is the subject of the remainder of this paper.

## Results and discussion

### Overview

In this section, we establish the nature of the discrepancy that leads to the UniFrac significance test producing different results on equivalent inputs. In order for two objects to be equivalent, we must show two things: first, that the two objects are isomorphic, and second, that both objects maintain the same semantic interpretation. Intuitively, two objects are isomorphic to each other if a procedure exists to transform one to the other and *back again*. A more mathematical definition of isomorphism follows in the next section.

In the preceding section, we described how trees input to the UniFrac program may or may not contain abundance counts. Below, we define a transformation for converting trees back and forth between these two formats. We briefly discuss how both tree formats have the same semantics. We then demonstrate that the UniFrac tool produces different outputs for the equivalent forms of the same input tree using a minimal example. Such a discrepancy is dangerous to researchers; it can lead them to false conclusions since their results may depend on the choice of input representation, making reproduction of results difficult. Finally, we illustrate the misleading results that can arise from this discrepancy using metagenomic data from a lake sediment study.

### Isomorphism of Trees With and Without Abundance Counts

**Figure 3:**
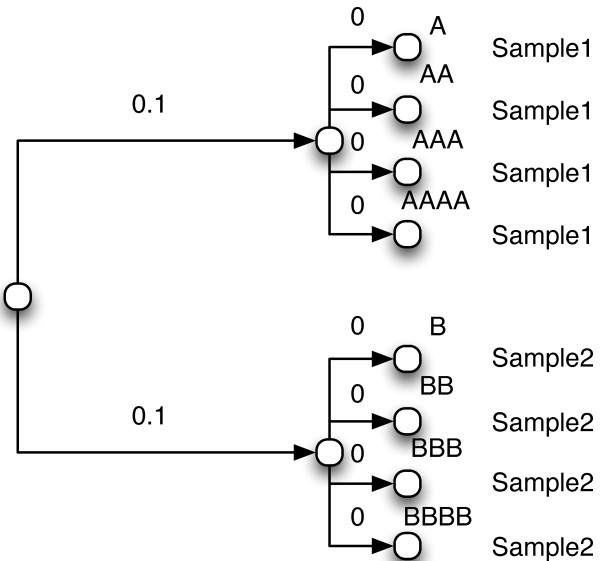
**Expanded input tree for UniFrac.** An expanded tree that is equivalent to that shown in Figure 2, but which does not use abundance counts.

We will show that the tree format that includes abundance counts is isomorphical to a tree with no abundance counts (or rather, where the abundance count associated with each node is assumed to be 1 and thus not shown). Here, we consider two objects *x* and *y* to be isomorphic to each other if and only if there exists a function *f* and its inverse *f*^-1^ such that *f*(*f*^-1^(*x*)) = *y* and *f*^-1^(*f*(*y*)) = *x*. Therefore, we will outline a simple, invertible transform *f* between the two tree formats. At each leaf node with either an abundance count *N*>1 for some sample *A* or with multiple sample labels, we create *N* new leaf nodes, which we then connect to the original leaf node (which is thus now an interior node) with a branch of weight 0. The sample label *SampleX* (for example *Sample*1 or *Sample*2 in Figures 2 and 3) is then associated with each of the new individual leaf nodes. If the original leaf node had more than one sample label, this is repeated for each label. Applying this transformation to the tree in Figure 2 results in the tree in Figure 3. The reverse transformation (*f*^-1^) is equally simple. First, we define a *pre-terminal* node as a node with only leaf nodes as children. For each pre-terminal node *T* for which all of *T*’s children are connected by a branch of length 0, we attach a count to *T* for each unique sample label *SampleN* associated with any child of *T*, where the count is the number of children of *T* that had label *SampleN*. We then eliminate all children of *T*, making *T* into a new leaf node. Clearly, by way of their construction, *f* is the inverse of *f*^-1^ and vice versa.

Since we now have a fully reversible transformation, we have satisfied the conditions to prove that the trees in Figure 2 and Figure 3 are isomorphic. Henceforth, we will refer to trees with abundance counts like the tree in Figure 2 as the *compact form* and larger trees like the one in Figure 3 as the *expanded form*.

### Semantics of compact and expanded trees

Both the compact and expanded form trees have the same semantics, and thus are valid input for the UniFrac significance test. In both cases, each leaf node represents an OTU and the count associated with it is an abundance count of that OTU (in the case of the expanded form, the counts are all implicitly 1). A distance value is attached to each branch; by means of their construction, these values are the same between both trees, except for the new branches in the expanded tree, which have an attached value of zero. Typically, these distances are expected to be edit distances between biological sequences, but the UniFrac formulation allows these values to be any distance metric. However, in mathematics it is standard that any function described as a distance function must satisfy a small number of very basic properties, one of which is the coincidence axiom, which states two objects have a distance of zero between them if and only if they are identical. Therefore, the semantics of a branch of zero length must necessarily be the same across both trees.

We have now shown that the compact and expanded form trees are both isomorphically *and* semantically equivalent and merely use a different visual representation. We would therefore expect any numeric calculations based on these trees to yield the same result.

### UniFrac P-Values on compact and expanded trees

We can now ask the question: what should be the result of a UniFrac significance test on the tree in Figure 3? Although we have only very briefly discussed how UniFrac distances are calculated, in the case of Figure 3 it is clear that all labelings other than the original (and of course the completely symmetric swap of the Sample1 and Sample2 labels) have an equal or smaller distance between the two samples than the original labeling itself. This is because the two longer, top level branches are at least partially shared by both samples in any scenario other than the original labeling, which will result in a smaller UniFrac distance. The p-value of the UniFrac significance test for this example is equal to the probability of randomly generating the original labeling. There are  possible ways to assign the labels to this tree, and since symmetric labelings are equivalent for our purposes (e.g. it doesn’t matter whether the first four or the last four nodes are all labeled with an A, so long as the As are all together), the probability of generating the original labeling at random is 2/70 or approximately 0.028.

We now upload the data for this tree (see Additional files 1 and 2) to the UniFrac web tool and perform a UniFrac significance test. We note that as the web tool by default generates 100 random labelings, we expect some variance in the p-values if the analysis is run multiple times, but we expect the range of the variance to be small. As expected, several trials performed by the authors yielded p-values in the range of 0 to 0.04.

Finally, we will compare this to the results for the tree in Figure 2. As we have previously shown, this tree is equivalent to the one in Figure 3, but because this tree is in compact form, the input files given to UniFrac are visually different from those for the tree in Figure 3 (contrast Additional files 3 and 4 with Additional files 1 and 2). For this tree, the resulting UniFrac significance value given by the web tool is 1.0 (and this result remains consistent no matter how many times we repeat this same input). This means that *all* randomly generated labelings had an equal or greater UniFrac distance between the two samples than in the original labeling. We can explain this discrepancy in output if we assume that, during the generation of random trees, the abundance counts are permanently associated with each sample label. In other words, in the case of Figure 2, whichever leaf node is associated with Sample1 automatically also has an abundance count of 4. Personal correspondence with the UniFrac authors has confirmed that is indeed the way the abundance counts are handled, and thus the discrepancy is not due to a code error nor to simple variance due to a relatively small number of random labelings. Because the compact-form tree has only two symmetric leaf nodes, it is irrelevant if the sample labels are swapped, and thus all randomly generated trees have the same UniFrac distance between samples as the original tree, resulting in a p-value of 1. Of course, this is completely different from the p-values in the range of 0 to 0.04 obtained for the tree in Figure 3, and so there is a discrepancy in output generated from semantically equivalent input.

We note that in this section, we have endeavoured to use the simplest possible example to illustrate the discrepancy between expanded and compact form trees. The problem is demonstrable with more complex trees as well.

### Other tools for comparing phylogenetic trees

We briefly investigated two other recent tools used for metagenomic analysis: Parallel-META [4] and Meta-Storms [5]. As Parallel-Meta does not directly compare comunities using phylogenetic trees, the disrepancy we describe above was not applicable. Meta-Storms does compare communities using phylogenetic trees in a manner similar to UniFrac, so we used Meta-Storms to analyze a simple artificial data-set. The data consisted of two environments, A and B, each containing 100 sequences. All sequences in environment A were identical to each other and all sequences in environment B were identical to each other, but completely different from the sequences in environment A. Meta-Storms reported that these two environments were *not* different from one another, mirroring the UniFrac result from the preceding section. Thus we conclude it is likely that Meta-Storms suffers from the same methodological problem that we identify in UniFrac above.

### Discussion on the discrepancy

The primary goal of this paper is to clearly demonstrate the discrepancy in output stemming from equivalent input for the UniFrac significance test. Our goal is not to make a definitive statement as to which output is better. However, here we will present some brief arguments and examples as to why the output produced when using expanded form trees as input may be more intuitive, scientifically useful, and robust.First, consider again the example in Figure 2, except now suppose that the abundance count for each leaf node is not 4 but 10,000, and that furthermore, the weight on each branch is not 0.1 but some much higher value, say 0.9. The p-value of a weighted UniFrac significance test on such an input tree is 1.0 for the same reasons we outlined in the preceding section, so the two communities would not be deemed significantly different. This seems an odd conclusion to draw, as this input tree represents two large, disjoint communities of individuals with a large phylogenetic distance between them. A significance test resulting from the expanded form of such a tree, on the other hand, yields a p-value of nearly 0, indicating that the two environments are indeed different with near-certainty.

Second, again using Figure 2 as a starting point, consider the case where the 4 individuals in Sample1 are not in fact identical, but are grouped together due to a clustering process (say, clustering DNA sequences at 97% sequence identity, a common operation in metagenomic studies), and similarly for Sample2. Let us then say that we wish to investigate the effect of this clustering by building a new tree where the sequences are not clustered at all. Such a tree would look very similar to the tree in Figure 3, except that the 0 weights on all the short branches would instead be some small, non-zero value (somewhere from 0 to 0.03 in this case). Note that because these branch weights are non-zero, this new tree is *not* a mere isomorphic transform of the original tree. However, a UniFrac significance test on this new tree would yield a p-value similar to the value obtained for the tree in Figure 3 (which was 0.028), and thus very different from the p-value of 1.0 for the tree in Figure 2. Thus, were we to use the result from the compact-form tree in Figure 2 as our starting point, we would conclude that the clustering process had a very large effect on determining community similarity, when in fact the clustering makes almost no difference if we start from the (equivalent) expanded form instead. This effect suggests that analysis of expanded form trees leads to more robust and consistent results in general.

### Effect of the discrepancy on sample data

We have observed this discrepancy to cause problematic results not just in the simple example described above, but also in a collection of our own DNA sequence data consisting of a metagenomic survey of lake sediment bacteria (manuscript in preparation). While a full description of these data is beyond the scope of this paper, we discuss the basics here. We used sediment samples from three different lakes; lake A is downstream from an industrial facility and lakes B and C are upstream reference lakes. Two different DNA extraction techniques were used to produce data from each sample, for a total of six samples. We would expect a priori that samples from the same lake would be similar to each other with the choice of DNA extraction having relatively little effect, and that lakes B and C would be more similar to each other than to lake A. Indeed, an examination of the weighted UniFrac pairwise distances between samples (see Table 1) agreed with this a priori expectation, as did other analyses we performed that are beyond the scope of this paper, but include statistical hierarchical clustering of OTUs, statistical comparison using AMOVA in the tool MOTHUR [6], visual inspection of OTU heatmaps and beta diversity metrics such as euclidean distances and unweighted UniFrac distances. However, p-values from the UniFrac Significance Test on this data, shown in Table 2, were contrary to all other analyses. It was in fact this observation that launched the investigation that resulted in this paper.

**Table 1:** **Normalized pairwise UniFrac distances on sample lake sediment data**

Sample	A1	A2	B1	B2	C1	C2
A1		0.09	0.33	0.35	0.31	0.33
A2			0.34	0.36	0.31	0.32
B1				0.10	0.15	0.19
B2					0.17	0.19
C1						0.10

Normalized, pairwise UniFrac distances between six samples of metagenomic lake sediment data. Samples A1 and A2 represent data from two different techniques used to extract DNA from a lake downstream of an industrial facility; samples B1 and B2 and C1 and C2 represent data from two upstream lakes.

**Table 2:** **Weighted UniFrac p-values on compact form of lake sediment data**

Sample	A1	A2	B1	B2	C1	C2
A1		0.03	0.05	0.02	0.13	0.22
A2			0.03	0.05	0.15	0.26
B1				0.06	0.49	0.44
B2					0.32	0.55
C1						0.48

Weighted UniFrac uncorrected p-values between six samples of metagenomic lake sediment data. As samples A1 and A2 are from the same lake, we would expect them to be similar, but the low p-value here indicates that there is a much higher probability that they are significantly different than, say, A1 and C2.

In this case, we appear to get more sensible results by performing the isomorphic transform we have described above prior to submitting data to UniFrac, and thus using the expanded form of our input. We provide a simple perl script for transforming data with abundance counts into its fully expanded equivalent (available at http://www.cs.usask.ca/research/research_groups/birl/unifrac_script.html). In Table 3 we show UniFrac Significance test p-values for our lake sediment data after running the transformation script. Many of the values in this table are zero, which is expected given the larger size of the expanded tree. However, the non-zero values are a much closer match to our a priori expectations than the p-values in Table 2.

**Table 3:** **Weighted UniFrac p-values on expanded form of lake sediment data**

Sample	A1	A2	B1	B2	C1	C2
A1		0.07	0.00	0.00	0.00	0.00
A2			0.00	0.00	0.00	0.00
B1				0.00	0.00	0.00
B2					0.00	0.15
C1						0.74

Weighted UniFrac uncorrected p-values between six samples of metagenomic lake sediment data using the expanded form as input. P-values are now much more consistent with the most important aspects of our expectations and other forms of analyses compared to the values in Table 2. For instance, the two downstream samples (A1 and A2) may be similar and are definitely different from all the upstream samples. Although there is a larger chance that B2 is similar to C2 than we might expect, the p-value for C1 and C2 is much higher still, which was not the case in Table 2.

## Conclusions

In this paper, we identify a discrepancy in the UniFrac methodology for testing of significant difference in community structure, showing how two equivalent inputs could generate vastly different outputs. UniFrac users need to be aware of this issue so as to avoid misleading and inconsistent results. We provide an example of the effect this discrepancy can have on real data. Finally, we provide software to perform the isomorphic transform so that data can be submitted to the existing UniFrac service in a consistent form.

## Methods

### Weighted UniFrac significance test with and without abundance counts

We generated p-values for Figures 2 and 3 using the UniFrac web service located at http://bmf2.colorado.edu/unifrac/index.psp. For Figure 2, Additional files 3 and 4 were used as the Newick tree and the Environment file, respectively. For Figure 3, Additional files 1 and 2 were used as the Newick tree and Environment file. In both cases, the "Select Analysis" option was set to "UniFrac Significance". The "Type of Test" option was set to "Each pair of environments". The "Number of Permutations" option was set to 100 (the default). The "Use Abundance Weights" option was set to "Yes".

## Electronic supplementary material

Additional file 1: **Newick Tree for Figure** 3 **.** Newick tree input to UniFrac web service for the example in Figure 3. (TXT 15 bytes)

Additional file 2: **Environment File for Figure** 3 **.** Environment file input to the UniFrac web service for the example in Figure 3. (TXT 51 bytes)

Additional file 3: **Newick Tree for Figure** 2 **.** Newick tree input to the UniFrac web service for the example in Figure 2. (TXT 65 bytes)

Additional file 4: **Environment File for Figure** 2 **.** Environment file input to the UniFrac web service for the example in Figure 2. (TXT 216 bytes)
